# The magnitude of neurocognitive impairment is overestimated in depression: the role of motivation, debilitating momentary influences, and the overreliance on mean differences

**DOI:** 10.1017/S0033291721004785

**Published:** 2023-05

**Authors:** Steffen Moritz, Jingyuan Xie, Danielle Penney, Lisa Bihl, Niklas Hlubek, Julia Elmers, Thomas Beblo, Birgit Hottenrott

**Affiliations:** 1 Department of Psychiatry and Psychotherapy, University Medical Center Hamburg-Eppendorf, Hamburg, Germany; 2 Centre Intégré Universitaire de Santé et de Services Sociaux de l'Ouest-de-l’Île-de-Montréal, Douglas Mental Health University Institute, Montreal, Canada; 3 Department of Psychiatry and Psychotherapy, Protestant Hospital Bethel, Bielefeld, Germany

**Keywords:** Assessment mediation, depression, motivation, neurocognition, test anxiety

## Abstract

**Background:**

Meta-analyses agree that depression is characterized by neurocognitive dysfunctions relative to nonclinical controls. These deficits allegedly stem from impairments in functionally corresponding brain areas. Increasingly, studies suggest that some performance deficits are in part caused by negative task-taking attitudes such as poor motivation or the presence of distracting symptoms. A pilot study confirmed that these factors mediate neurocognitive deficits in depression. The validity of these results is however questionable given they were based solely on self-report measures. The present study addresses this caveat by having examiners assess influences during a neurocognitive examination, which were concurrently tested for their predictive value on performance.

**Methods:**

Thirty-three patients with depression and 36 healthy controls were assessed on a battery of neurocognitive tests. The examiner completed the Impact on Performance Scale, a questionnaire evaluating mediating influences that may impact performance.

**Results:**

On average, patients performed worse than controls at a large effect size. When the total score of the Impact on Performance Scale was accounted for by mediation analysis and analyses of covariance, group differences were reduced to a medium effect size. A total of 30% of patients showed impairments of at least one standard deviation below the mean.

**Conclusions:**

This study confirms that neurocognitive impairment in depression is likely overestimated; future studies should consider fair test-taking conditions. We advise researchers to report percentages of patients showing performance deficits rather than relying solely on overall group differences. This prevents fostering the impression that the majority of patients exert deficits, when in fact deficits are only true for a subgroup.

## Introduction

Reviews and meta-analyses (Bortolato, Carvalho, & McIntyre, 2014; Goodall et al., 2018; Klimkeit, Tonge, Bradshaw, Melvin, & Gould, 2011; Lee, Hermens, Porter, & Redoblado-Hodge, 2012; Parkinson, Rehman, Rathbone, & Upadhye, 2020; Rock, Roiser, Riedel, & Blackwell, 2014; Snyder, 2013) suggest that patients with depression display neurocognitive dysfunctions across a wide range of domains relative to healthy controls. Neurocognitive deficits in turn seem to mediate functional outcome, such as work performance (Cambridge, Knight, Mills, & Baune, 2018; Evans, Iverson, Yatham, & Lam, 2014; McIntyre et al., 2013). Impairments in neurocognitive performance are traditionally ascribed to deficits in functionally corresponding brain areas (for studies in depression linking neuropsychological and brain functioning see Ge et al., 2019; Liu et al., 2020; Milak et al., 2019; Yan et al., 2019) and it is thus common in clinical neuropsychology to denote a test (battery) as either being sensitive to a certain brain region (e.g. Gualtieri and Johnson, 2008) or even equate the two. Hence, memory tests are sometimes referred to as ‘temporal lobe tests’ (Eisenberg & Levin, 1989) and tests of executive functioning as ‘frontal tests’ (Cox et al., 2014; Kopp et al., 2013). This mingling of tests and brain functioning stems from a time when neuroimaging techniques were invasive, dangerous, or not widely available, and when neuropsychological tests such as the Benton Test (Benton, 1945) served as proxies of brain dysfunction. Indeed, those with brain injury reliably perform worse than controls according to meta-analyses (Dunning, Westgate, & Adlam, 2016; Königs, Engenhorst, & Oosterlaan, 2016). In the remainder of this article, we will pursue the question of whether the assumption that neuropsychological deficits are primarily due to impairment in cortical regions governing neurocognition is valid or not for depression.

As was previously argued (Moritz et al., 2017c), the notion that depression is related to and allegedly even caused by cortical dysfunction likely fosters ‘brain stigma’. While a biological (biogenetic) model may decrease self-blame in some individuals (however see Kemp, Lickel, and Deacon, 2014), it fuels prognostic pessimism, social exclusion, and helplessness (Lebowitz & Appelbaum, 2019; Speerforck, Schomerus, Pruess, & Angermeyer, 2014), which in turn can negatively impact health care utilization (Schnyder et al., 2018). Another possible consequence is ‘dementia worry’ (Kessler, Südhof, & Frölich, 2014). A reanalysis of a recent survey (Miegel, Jelinek, & Moritz, 2019) indicated that one in eight patients with depression or obsessive–compulsive disorder (OCD) endorsed that OCD or depression cannot be treated effectively with psychotherapy because it is a brain disorder.

The emphasis on cortical alterations in depression and its consequences (e.g. ‘brain stigma’) might be tolerable if they were undeniably true. Yet, there is increasing reason to doubt a simple deterministic relationship between neurocognitive and cortical alterations in depression. While we do not deny that neurocognitive deficits are present in a large subgroup of patients, it is worth noting that not all trials have detected neurocognitive (Biringer et al., 2007; Clark, Kempton, Scarnà, Grasby, & Goodwin, 2005) or social cognitive (Fieker, Moritz, Köther, & Jelinek, 2016) deficits in (remitted) depression. An Australian study found no differences between patients with depression and healthy controls on 17 out of 18 parameters; only one parameter of attention set-shifting was significantly worse in the patient group (Purcell, Maruff, Kyrios, & Pantelis, 1998). A meta-analysis on young people suffering from depression aged 12–25 years (Goodall et al., 2018) reported that deficits in processing speed/reaction time and verbal learning disappeared when the methodological quality of studies was accounted for. In addition, these deficits are unlikely to be specific to depression and their causal mechanism thus elusive (East-Richard, Mercier, Nadeau, & Cellard, 2020). In fact, neurocognitive deficits are well-established for a range of psychiatric disorders and the specificity of impairment in single domains is poor (Abramovitch, Short, & Schweiger, 2021).

A growing body of evidence indicates that test impairment in depression is related to potential mediators such as (test) anxiety (Dorenkamp & Vik, 2018; Kizilbash, 2002), worry (de Vito, Calamia, Greening, & Roye, 2019), and poor effort (Benitez, Horner, & Bachman, 2011). Scheurich et al. (2008) highlighted the role of motivational deficits by showing that with the application of goal-setting instructions, depressed patients and control participants achieved similar memory performance. Yet, not all studies have confirmed such a relationship (Beblo, Driessen, & Dehn, 2020). Other research (Crane, Barnhofer, Visser, Nightingale, & Williams, 2007; Grant, Mills, Judah, & White, 2021; Joormann & Gotlib, 2008; Schwert, Aschenbrenner, Weisbrod, & Schröder, 2017; Watkins & Roberts, 2020; Whitmer & Gotlib, 2012) suggests that rumination compromises cognitive performance in patients with depression (however see also Vălenaș and Szentágotai-Tătar, 2017). Patients with depression also seem to avoid subjectively complex cognitive tasks despite intact ability (Bowie, Milanovic, Tran, & Cassidy, 2016). Recently, we tested the hypothesis that neurocognitive assessments, which are routine in many psychiatric facilities, evoke stress in individuals with depression, likely compromising subsequent performance. In line with our hypothesis, we observed that patients with depression were more fearful of test outcomes, less motivated (based on a retrospective assessment), and complained more about negative momentary influences than controls when assessed using a newly developed self-report questionnaire, the Momentary Influences, Attitudes and Motivation Impact on Cognitive Performance Scale (MIAMI; Moritz et al., 2017c). When MIAMI scores were entered as a covariate, group differences were largely reduced, and the MIAMI proved a significant mediator in three out of four analyses. Similar results have been found for OCD (Moritz, Hauschildt, Saathoff, & Jelinek, 2017a), schizophrenia (Moritz et al., 2017b, 2020), and alcohol use disorder (Moritz et al., 2018).

Objective impairments often manifest as subjective cognitive complaints by patients (Lahr, Beblo, & Hartje, 2007). Such complaints are often taken as a proxy for objective deficits (e.g. Reid and MacLullich, 2006, p. 471), particularly when tests are not available. However, subjective complaints show poor correspondence with real deficits (Dhillon, Videla-Nash, Foussias, Segal, & Zakzanis, 2020; Gass & Patten, 2020; Keilp et al., 2018) but are closely linked to depressive symptoms (Balash et al., 2013; Moritz, Ferahli, & Naber, 2004; Slavin et al., 2010). Subjective complaints about one's neurocognitive decline seem to reflect the depressive symptom of self-devaluation (Lahr et al., 2007), where the individual negatively appraises virtually all of his or her abilities/characteristics, including neurocognitive functioning.

### The present study

The aforementioned pilot study on the MIAMI scale in depression (Moritz et al., 2017c) was compromised by the administration of a self-report questionnaire; one may argue that patients with depression may not be fully able to objectively assess their symptoms (i.e. lack of cognitive insight) and that strategic motives may have distorted responses (e.g. endorsing poor motivation and/or psychological well-being as an excuse for malperformance). In lieu of using the MIAMI scale, the present study asked examiners to observe different aspects deemed relevant for performance, such as motivation, test anxiety, and distracting symptoms (e.g. rumination) during the assessment. Using a preliminary version of the self-developed present questionnaire termed Experimenter Performance Assessment, the negative effect of symptoms and motivation on performance was confirmed in OCD (Moritz et al., 2017a).

As in the previous study, we analyzed data using mediation analysis and analyses of covariance (ANCOVAs). We hypothesized that group differences on neurocognitive performance would be significantly reduced when psychological factors such as poor motivation and distraction due to symptoms are accounted for. We also asked 18 professionals with practical experience in neuropsychology to rate the reciprocity of neurocognitive functioning and the items of the newly devised questionnaire. In doing this we aimed to eliminate items that would reflect an epiphenomenon of neurocognitive impairment.

## Methods

### Participants

We recruited 33 patients with a diagnosis of unipolar depression according to the ICD-10 and DSM-5. Most participants were inpatients at the Department of Psychiatry and Psychotherapy at the University Medical Center Hamburg (Germany). Patients were assessed as part of a routine psychiatric assessment, which is more often requested if neurocognitive deficits are suspected (see discussion). The primary diagnosis of depression as well as comorbid disorders was determined by the clinician in charge, either physicians or psychologists, who also completed the Brief Psychiatric Rating Scale (BPRS) as a measure of overall symptom severity (Overall & Gorham, 1962). The majority of patients were medicated with antidepressant agents (*n* = 26); nine patients were prescribed an antipsychotic agent, and eleven (occasionally) received tranquilizers. Bipolar disorder, schizophrenia/psychosis, substance abuse, autism, and major neurological disorders of the central nervous system (e.g. multiple sclerosis, stroke) were exclusion criteria, as was depression secondary to OCD and post-traumatic stress disorder. Other diagnoses such as comorbid anxiety were tolerated.

Patients were compared to 36 healthy controls. The absence of psychiatric disorders was confirmed via the Mini International Neuropsychiatric Interview (MINI version 7.0.2 for DSM 5; Sheehan et al., 1998). Control participants were recruited via word-of-mouth and advertisements. General exclusion criteria for both groups were major neurological disorders of the central nervous system (e.g. multiple sclerosis) and age below 18 or above 65 years. The study was approved by the ethics committee of the German Psychological Association (DGPS, EK122016). All participants provided written informed consent prior to participating in the study. The groups did not overlap with the sample of the initial study.

### Neuropsychological assessment

All assessors received 6 weeks of rigorous training and their adherence to instructions was confirmed by an experienced neuropsychologist prior to testing.

### Trail-making test A and B (TMT) – psychomotor speed and set-shifting

Psychomotor speed was assessed with the TMT Part A (adult version) (Reitan, 1992). The TMT-A requires the individual to connect numbers in ascending order as quickly as possible, whereas Part B captures set-shifting and requires the participant to alternate between numbers and letters, again in ascending order. Age-adapted standard scores were applied (Tombaugh, 2004).

### Wisconsin card sorting test (WCST) – executive functioning

Executive functioning was assessed with a computerized version of the WCST (Heaton, 1981; Loong, 1990). The procedure closely follows the original non-computerized test. The participant was shown a maximum of 128 cards, which had to be matched according to three varying sorting principles (i.e. number of items, color, shape), which were unknown to the individual. Via a high or low tone and a corresponding verbal cue, feedback was given on the correctness of each match. Categories completed and perseverative errors served as dependent variables.

### Auditory verbal learning test (AVLT) – memory

Verbal memory and learning were assessed with the German version of the AVLT (Helmstaedter, Lendt, & Lux, 2001; Lezak, 1995). A list of 15 words (List A) was read to the participant five times. After each trial, as many words as possible had to be repeated in loose order. After five trials, the individual had to memorize words from a separate inference list (List B). Then, words from List A had to be recalled again without renewed presentation. Thirty minutes later, words had to be repeated from List A only. Learning was measured by the sum of correctly recalled words on trials 1 through 5. The number of correctly recalled words after the 30-min delay served as an index for long-term memory. Normative scores for the German version of the task are available for different age ranges (Helmstaedter et al., 2001).

### d2-R test – selective attention

The d2-R test is a letter cancellation test that measures selective attention (Brickenkamp, Schmidt-Atzert, & Liepmann, 2010). Following a practice trial, 14 rows containing target and distractor stimuli were presented. The participant had to cross out the letter d whenever it was presented with two small lines; d's with more or less than two lines, or any stimuli containing the character p, represented distractors. Participants had 20 s for each row. The test is scored according to a number of correctly crossed out stimuli and errors. Normative scores for the concentration performance (‘Konzentrationsleistung’) parameter (KL) were determined (for this parameter only rows 2–13 are considered). Age-adjusted normative scores exist from a large population sample (Brickenkamp et al., 2010).

### Story recall from the Wechsler memory scale (WMS) – revised edition – memory

Two brief stories were read to the participant. Immediately following each story, the participant had to repeat as much of the story as they could remember (short-term memory) (Woodard & Axelrod, 1987). Thirty minutes later, the participant again had to recall as much of the story as possible (long-term memory). No interfering verbal memory tests were presented during the retention interval. German norm values were applied (Härting et al., 2000).

### Subtests from the Wechsler adult intelligence scale, 4th edition (WAIS-IV) – reasoning and visuospatial performance

Two subtests of the WAIS-IV were administered, which is a test battery for global intelligence. Scaled scores from a large German population sample were applied (Petermann, 2012; Wechsler, 2008).

#### Matrix reasoning

The matrix subtest measures nonverbal reasoning. The individual was presented with a pattern sequence and had to select the item that completed the sequence from five alternatives.

#### Block design

In this visuospatial performance test, the participant had to match colored cubes to a two-dimensional pattern as quickly as possible. Task difficulty increased over time. Scoring was made according to both accuracy and time.

### Impact on performance scale

During the neurocognitive assessment, the examiner rated patients' performance, behavior, and emotional responses using the Impact on Performance Scale (see appendix). The scale is an extended version of the Experimenter Performance Assessment, which was previously administered to a sample of OCD patients (Moritz et al., 2017a). The first part consisted of seven items for which the exact frequency of occurrence was noted (the examiner inconspicuously made a tick mark whenever a certain behavior was exhibited, for example playing with mobile phone/taking incoming calls), which may also aid assessment of the subsequent 22 items. The remaining items evaluated test anxiety, impairment/distraction due to symptoms (e.g. rumination), unfavorable contextual influences (e.g. tiredness), and motivation (both poor and high). Ratings were made on a four-point scale ranging from ‘applies fully’ to ‘does not apply at all’. The rater also noted when an item could not be assessed. The final two items related to compliance (applicable/not applicable). To rate the Impact on Performance Scale, the examiner had to rely on the participant's behavioral manifestations and utterances during the assessment (e.g. comments such as ‘This is boring’ or ‘Do we really need to do this?’ may be considered indicators of poor motivation; self-degrading remarks or reassurance-seeking may be indicators of rumination). The internal consistency of the present version is Cronbach's *α* = 0.76 (beta-version: 0.8).

The subscale algorithm was derived from a factor analysis of items 1–22 (the last items were discarded due to their binary format). Data from 132 psychiatric patients (including the present sample) who underwent neuropsychological testing were submitted to varimax-rotated factor analysis. Items on delusional ideas and compulsions during testing (items 7 and 8) were excluded due to lack of variance (items were never endorsed). The Kaiser-Meyer-Olin score was 0.80 and Bartlett's test of sphericity was significant, χ^2^ (190) = 807.49, *p* < 0.001. Five dimensions showed an eigenvalue greater than 1 and explained 62.85% of the variance. Scree-plot inspection suggested a two-dimensional solution explaining 41.34% of the variance. The first dimension, entitled *Well-Being During Assessment*, captured items such as fear to make mistakes, performance anxiety, reassurance-seeking, tension, and other negative feelings in response to either the test material or the situation. Higher subscale scores indicate greater wellbeing. The second factor, termed *Motivation*, related to motivation, lack of concentration, boredom, and fatigue. Again, higher subscale scores denote greater motivation.

Based on the loading matrix shown in Table 1 we computed two subscales considering items that loaded at least 0.5 on one factor (the difference to the other factor was set as at least |0.2|). The internal consistency of the 22 main items was Cronbach's *α* = 0.79. To assess content validity, we asked 18 psychologists with practical experience in neuropsychology (most had a degree in neuropsychology awarded by the German Neuropsychological Society, GNP) to indicate which of the variables captured in the questionnaire would likely or possibly reflect poor neuropsychological functioning rather than being a confounding contributor to performance.

**Table 1.** Group differences on sociodemographic background characteristics and scores on the Impact on Performance Scale

Variables	Depression (*n* = 33)		Healthy (*n* = 36)		Statistics			
Demographic characteristics	*M*/[*n*]	*s.d.*/[%]	*M*/[*n*]	*s.d.*/[%]	*|t|*/[χ^2^]	df	*p*	*|d|*
Sex (female/male)	[14/19]	[42%/58%]	[19/17]	[53%/47%]	0.74	1	0.390	–
Age in years	42.82	13.80	43.86	14.03	0.31	67	0.757	0.07
Years of formal education	11.39	1.69	11.43	1.65	0.11	65	0.916	0.02
BPRS total score	43.50	10.70	–	–	–	–	–	–
BPRS depression score	3.31	(1.70)	–	–	–	–	–	–
*Impact on performance scale*								
Well-being during assessment	2.90	0.79	3.45	0.49	3.44	52.85	0.001	0.84
Motivation	3.42	0.59	3.60	0.38	1.54	54.09	0.128	0.36
Total score	3.16	0.61	3.52	0.35	3.04	50.51	0.004	0.72

BPRS, Brief Psychiatric Rating Scale.

### Strategy for data analysis

The sample size allowed for the detection of medium-to-large effect size (as calculated with g*power) between the patient and healthy control group in accordance with the reviews cited in the introduction.

In line with prior studies, no clear neurocognitive profile was expected for patients with depression. We composed an overall neurocognitive index, which aggregated all speed (i.e. the Trail-Making Test scores) and performance parameters (e.g. learning) displayed in Table 2. Scores of these parameters were z-transformed, with high scores indicating better performance (i.e. greater accuracy and performance speed; some parameters had to be reversed accordingly). Similar to prior studies, we first compared the two groups using independent samples *t* tests on all neurocognitive parameters. To examine mediators on neuropsychological performance across group differences, we adopted a two-fold strategy. We calculated a mediation analysis using Hayes' process procedure (Hayes, 2013), specifically model 4 with 5000 bootstrap samples. Group status (depression *v.* healthy) served as the independent variable (x), the overall neuropsychological functioning as the dependent variable (y) and the total score of the Impact on Performance Scale as the mediator (M). In addition, we calculated analyses of covariance by entering the total score of the Impact on Performance Scale as a covariate to examine whether effect sizes would decrease substantially; the latter analyses aimed to aid the interpretation of the primary mediation analysis.

**Table 2.** Differences in neurocognitive functioning between healthy and depressed individuals

Variables/Domains	Healthy (*n* = 36)			Depression (*n* = 33)			% 1SD ⩽ Norm [% 2SD ⩽ Norm]	Group differences				ANCOVA (covariate: Impact on Performance Scale)	
	*M*	*s.d.*	Norm	*M*	*s.d.*	Norm	%	*|t|*	df	*p*	*|d|*	*p*	*|d|*
*Speed*													
Trail-Making Test A in sec.	25.06	8.96	63.83^P^	36.82	20.89	42.30^P^	8.3%/27.3% [n.a.]	2.990	42.603	0.005	0.73	0.053	0.48
*Attention*													
d2-R concentration index	145.63	32.97	45.80^P^	125.24	36.11	29.45^P^	17.1%/42.4% [n.a.]	2.434	66	0.018	0.59	0.122	0.39
*Memory*													
AVLT learning	58.08	7.85	57.31^T^	51.39	12.28	51.30^T^	2.8%/18.2% [n.a.]	2.719	67	0.008	0.65	0.029	0.54
AVLT retention	12.19	2.74	53.75^T^	11.19	3.07	48.24^T^	11.1%/18.8% [n.a.]	1.427	66	0.158	0.34	0.350	0.23
Logical Memory immediate	32.42	6.09	70.58^P^	23.56	8.61	36.19^P^	5.6%/38.7% [0.0%/0.0%]	4.938	66	<0.001	1.19	0.001	0.91
Logical Memory delayed	29.08	7.11	69.75^P^	20.06	9.87	35.11^P^	8.3%/50% [0.0%/0.0%]	4.333	65	<0.001	1.05	0.004	0.75
*Reasoning*													
Matrix Reasoning	19.14	4.84	9.89^s^	20.12	3.57	10.55^s^	16.6%/6.1% [8.3%/0%]	0.953	67	0.344	0.23	0.018	0.60
*Spatial Performance*													
Block Design	48.28	11.22	10.53^s^	43.68	12.91	9.39^s^	16.7%/32.2% [2.8%/3.2%]	1.561	65	0.123	0.38	0.758	0.07
*Executive Functioning*													
Trail-Making Test B in sec.	62.65	28.18	59.44^P^	85.62	51.10	40.53^P^	16.7%/33.3% [n.a.]	2.338	67	0.022	0.56	0.325	0.25
WCST Categories	5.34	1.19	–	4.58	2.09	–	–	1.788	46.198	0.080	0.45	0.598	0.12
WCST Perseveration in %	13.40	6.63	–	16.52	12.88	–	–	1.257	64	0.213	0.30	0.833	0.08
Aggregated neurocognitive score (z-transformed)	0.24	0.52	–	−0.27	0.69	–	–	3.45	67	0.001	0.84	0.047	0.49

T, T-scores (*M* = 50); P, percentile (*M* = 50); S, scaled score (*M* = 10); n.a, not available.

## Results

### Group differences on baseline variables and neurocognitive functioning – uncorrected

No differences occurred for any demographic characteristics between the two groups (Table 1). On average, patients showed mild depressive symptoms. Patients differed significantly from controls on the *Well-Being During Assessment* subscale and the total score but not the *Motivation* subscale of the Impact on Performance Scale.

As can be derived from Table 2, individuals with depression performed significantly worse than controls on 6 out of 11 neuropsychological test parameters; two of the significant differences were large (*d* ⩾ 0.8), and four were medium (*d* ⩾ 0.5). Group differences for the aggregated score revealed a large effect size (*d* = 0.84).

### Deviations from normal performance (application of standardized scores)

A total of 29.67% of patients showed impairment (mean) compared to only 11.47% in healthy individuals (median: 32.75% *v.* 13.75%) as reflected by norm scores. For two tests, normative data for the performance of at least two standard deviations below the mean was available; these rates were, however, low for patients (AVLT learning and retention: 0%; Matrix: 0%; Block Design: 3.2%). The average performance of the depressed group was never in the range of one standard deviation below the mean (mainly lower normal scores were achieved). For controls, performance as a group was in the upper normal range, particularly for AVLT learning, where significant group effects were rather owing to high performance in the control group than abnormal performance in patients (T-score: 57.31 *v.* 51.30).

### Association between impact on performance scale and neuropsychological functioning

For 5 out of 6 analyses, the aggregated neurocognitive score was significantly correlated with the *Well-Being During Assessment* subscale (depressed: *r* = 0.431, *p* = 0.012; healthy: *r* = 0.282, *p* = 0.096), the *Motivation* subscale (depressed: *r* = 0.672, *p* < 0.001; healthy: *r* = 0.357, *p* = 0.033) and the total score (depressed: *r* = 0.604, *p* < 0.001; healthy: *r* = 0.389, *p* = 0.019) of the Impact on Performance Scale.

### Group differences on neurocognitive functioning – corrected

**Fig. 1.**
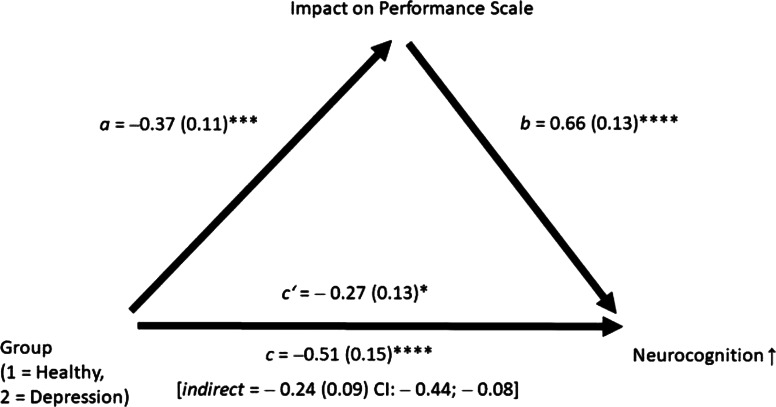
Mediation analysis. The indirect effect was significant owing to a large discrepancy between the total and direct effect. *p < 0.05; **p < 0.01; *** *p* < 0.005, **** *p* < 0.001.

The relationship between group and neurocognition (i.e. the aggregated neurocognitive score) was mediated by the total score of the Impact on Performance Scale scores (Fig. 1) as the confidence intervals (CIs) of the indirect effect did not cross zero (5000 bootstrap samples). The direct effect (*p* < 0.001) was largely reduced but remained significant (*p* = 0.048). We reran the analysis by removing three items that the majority of the 18 experts suspected to reflect poor neurocognitive functioning rather than being a confounding contributor to performance (items 12, 20, 22 – these items do not fully discern the causal direction between malperformance and the alleged mediator; item 13 was also deemed critical but not part of the total score; see appendix). Results remained essentially unchanged (the lower and upper limit did not cross zero, – 0.20 (standard error: 0.13) [CI: – 0.39 to – 0.05]), suggesting a significant indirect effect.

In line with the mediation analysis, the effect sizes of the group differences for single parameters were significantly reduced when the Impact on Performance Scale was entered as a covariate; 3 of 11 comparisons remained significant with only 1 yielding a large effect size (*d* ⩾ 0.8). In one parameter (matrix reasoning), the depressed individuals now performed significantly better than controls. On average, the aggregated neurocognitive score was reduced to a medium effect size.

Finally, we created four neurocognitive domains following DSM-5 definitions (Sachdev et al., 2014). For memory (AVLT, logical memory), complex attention (TMT-A, d2), perceptual-motor function (matrix reasoning, block design), and executive functioning (WCST, TMT-B), mediation was confirmed; none of the CIs crossed zero (memory: −0.16 (standard error: 0.10), CI −0.38 to −0.0025 (four decimals are shown to confirm that zero is not crossed); complex attention: −0.22 (0.11), CI −0.47 to −0.04; perceptual-motor function: −0.31 (0.13), CI −0.60 to −0.11; executive function: −0.31 (0.11), CI –0.54 to –0.11).

## Discussion

### Impetus of the study

It seems almost textbook knowledge that with depression comes large neuropsychological impairment (see textbooks on biological psychiatry by Panksepp, 2004; Trimble and George, 2010). Some more recent studies however have raised concerns against this claim, attributing neurocognitive test impairment at least partly to mediators such as poor motivation or distraction caused by rumination. Moreover, the deficits found in depression also manifest in other psychiatric disorders (Abramovitch et al., 2021) challenging a direct role in the specific pathogenesis of depression. The present study examined contextual and personal mediators of malperformance. It also addressed a reasonable objection to a prior study (Moritz et al., 2017c) that had used a self-report measure to assess symptoms, which may not be fully valid due to a lack of cognitive insight in patients.

### Summary of results

At first glance, the present results tie in well with the notion of large neurocognitive impairment in depression. As expected from reviews and meta-analyses (Bortolato et al., 2014; Goodall et al., 2018; Klimkeit et al., 2011; Lee et al., 2012; Parkinson et al., 2020; Rock et al., 2014; Snyder, 2013), the depressed sample performed worse than nonclinical controls on the majority of parameters. For the aggregated neurocognition score the difference to controls achieved a large effect size. However, impairments, as defined by one standard deviation below the mean, were seen in less than one-third of patients.

As hypothesized, patients with depression scored worse than controls on the total score of the Impact on Performance Scale, which is mainly attributable to the *Well-Being During Assessment* subscale tapping into concerns about the assessment, such as fears about the poor outcome and unfavorable momentary influences. Scores for the *Motivation* subscale were numerically lower with a small-to-medium effect size but did not achieve significance compared to controls. The latter finding corroborates a prior finding (Moritz et al., 2017c) where motivation was only lower for one of two scores (see also Beblo et al., 2020). In the present study, the Impact on Performance Scale total score mediated the relationship between group status and neurocognition, and the direct effect (Group – neurocognition) now barely reached statistical significance (*p* = 0.048). An indirect effect was confirmed for core neuropsychological subdomains (memory, complex attention, perceptual-motor function, executive function). This was mirrored by ANCOVA results, where effect sizes were largely attenuated from a large effect for the aggregated score to a medium effect. For one parameter, the matrix test, depressed patients now performed significantly better than controls. For many neuropsychologists, true impairment starts at two standard deviations below the mean (Abramovitch & Schweiger, 2015). We, therefore, looked at results from scores, where such information was available. For three parameters (two memory parameters as well as matrix reasoning), 0% of depressed patients scored two standard deviations below the mean, while 3.2% scored two standard deviations below the mean on block design.

An inspection of norm scores, even before adjusting for mediators, indicates that group differences were perhaps inflated by our choice of controls. While matched on sex, age and education, our nonclinical participants were without psychological disorders and thus not representative of the general population, which would also include people with psychological and somatic problems who may display some test deficits. For some parameters, controls performed numerically in the upper range of normality while patients' scores were in the lower range of normality (numerically above average on Matrix reasoning and AVLT learning). Group differences should thus not be mistaken as indicators for the presence of normal *v.* abnormal scores.

As discussed before (Moritz et al., 2017c), some contributors to poor test performance such as distraction caused by rumination, lack of sleep, or anhedonia may mirror core symptoms of the primary disorder and cannot be easily controlled for. Still, these should be considered as confounds because meaningful inferences from neuropsychological tests to brain-related impairment can only be drawn if participants perform to the best of their potential.

### The implications of a biological and psychological model of neurocognitive deficits for self-perception and treatment

Our study also aimed to account for a known chicken-or-the-egg problem in neuropsychology relating to the potentially reciprocal relationships between neuropsychological deficits and alleged mediators such as poor motivation. When we considered only those items capturing true mediators by means of expert ratings (i.e. capturing a unidirectional relationship), the primary result was essentially replicated.

The relationship between depression and neurocognition is complex, and earlier reports of a direct relationship between symptom severity and neuropsychological functioning (McDermott & Ebmeier, 2009) have been recently challenged (Keilp et al., 2018). Apart from the mediators discussed in the present paper, we should be prepared to find that neurocognition may contribute to depression not directly but via intermediate factors, especially academic achievement (Mayes, Calhoun, Bixler, & Zimmerman, 2009), which is predictive of work status and thus also impacts social rank. Clearly, neurocognitive deficits attenuate educational performance and thus impact job opportunities, potentially leading to economic hardship (see also Lorant, 2003; Heflin and Iceland, 2009).

From a practical standpoint, one may ask to what extent it matters whether neuropsychological functioning is attributable to specific disruptions in neurobiological functioning, or in part due to psychological processes associated with depression. Either way, depressed individuals do not perform as well on behavioral/ ‘objective’ measures of cognitive functioning relative to non-depressed individuals. Importantly, these impairments likely carry over into everyday life. We believe that this is an important question/distinction for two reasons. First, it casts doubt on the notion that depression is a purely neurobiological disorder marked by fundamental neurocognitive deficits, which may attenuate some of the stigma associated with the disorder. Second, if neurocognitive deficits can be attributed in part to test anxiety, for example, it suggests that patients may require other types of treatment targeting processes such as poor motivation or fatalism. Finally, we have proposed recommendations on how to treat deficits (depending on their respective causes) in individuals with schizophrenia who also display poor test results (Moritz et al., 2020). Whether such recommendations are relevant to depressed patients awaits to be tested.

### Limitations

Several limitations of our study need to be acknowledged. First, the sample size was small, and was collected at only one site. Most patients were medicated; thus, investigating the impact of antidepressant drugs on performance was not possible. Moreover, diagnoses and comorbid disorders were not determined by a (semi)structured interview. There was also a deficit-leaning selection bias given neuropsychological testing is often requested in routine assessment for patients with suspected deficits. As such, the level of neurocognitive impairment in the present sample was likely larger than in a representative sample. Multi-center studies are desirable in the future as contextual influences may vary across settings – for example the examiner's attitude/feedback towards a patient may attenuate, augment or elicit mediators like test anxiety and motivation (Murphy, Michael, Robbins, & Sahakian, 2003). While our questionnaire relied on external ratings and considered more factors than our self-report scale, further aspects should be taken into account. For example, perceptual deficits, stereotype threat, defeatist beliefs, and physical inactivity in addition to somatic factors such as hypertension may compromise performance; such relationships have been already confirmed in other disorders (for a review see Moritz et al., 2020) and deserve further examination in depression. For future studies, we also recommend discerning between state and trait factors, that is, examining factors that are evoked specifically by the assessment situation (e.g. test anxiety) and general factors unrelated to test taking.

Finally, we recommend implementing additional effort tests and longitudinal assessments to pinpoint causal mechanisms.

## Conclusions

Our study suggests that meta-analyses implicating large neurocognitive deficits in depression may oversimplify a more complex relationship and contribute to the ‘brain stigma’ associated with depression. First, while it is not wrong to present group differences, solely reporting means likely obscures the real prevalence of deficits in this population, which may both be under- or overestimated (Gualtieri & Morgan, 2008). We recommend also reporting the percentage of patients with deficits in the abstract of the publication to clarify if impairments are ubiquitous or concern only a subgroup, just as in our study where the magnitude of mean impairment was large but was caused by only a minority of patients. Importantly, researchers should reconsider whether conservatively selected healthy controls are the best control group when estimating the degree of impairment. Removing those with psychopathological symptoms, which are common in non-psychiatric samples, may create ‘super-controls’ who further increase group difference; samples drawn from the general population may more accurately represent a fair control group. Second, confounds also need to be considered. Rather than the monocausal attribution of neurocognitive deficits to dysfunctions in functionally corresponding brain areas, secondary effects need to be considered and results adjusted accordingly. While we do not deny the role of biological factors and think that psychological and biological models are not counter-exclusive, an overestimation of neurocognitive deficits likely fosters biological (‘medicalization’) models of depression, which according to meta-analytic evidence, have a detrimental effect on psychological well-being (Kvaale, Haslam, & Gottdiener, 2013). To this end, self-help treatments developed by our group have begun to address brain stigma and its consequences in patients with OCD and depression (Moritz, Bernardini, & Lion, 2020; Moritz, Irshaid, Beiner, Hauschildt, & Miegel, 2019b). Finally, basic researchers, although often not directly involved in treatment, should adhere to the Hippocratic Oath of *non nocere* (‘to abstain from doing harm’); we should only infer true neurocognitive deficits when alternative sources can be ruled out.

While the conventional treatment of neurocognitive deficits is cognitive remediation, future research should test whether the neurocognitive performance of patients with depression is improved by addressing emotional and motivational mediators (e.g. self-stigma, performance anxiety). We must also gain more knowledge regarding the real-world implications of neurocognitive deficits in view of some reports suggesting only low to moderate ecological validity (Van Der Elst, Van Boxtel, Van Breukelen, & Jolles, 2008).

## Data Availability

The data that support the findings of this study are available from the corresponding author upon reasonable request.

## Supplementary material

For supplementary material accompanying this paper visit https://doi.org/10.1017/S0033291721004785. click here to view supplementary material
