# Supplementary material for: The magnitude of neurocognitive impairment is overestimated in depression: the role of motivation, debilitating momentary influences, and the overreliance on mean differences
Source: Psychol Med. 2022 Jan 13;53(7):2820–30. doi: 10.1017/S0033291721004785 (PMC10235659; doi:10.1017/S0033291721004785)
Supplement: Supplementary file 1 [file S0033291721004785sup001.docx]

Appendix: Recording Form for use during testing * - Supervisor

| **Events** | **Frequency** |
| --- | --- |
| Yawns |  |
| Interruptions/pauses requested by the participant or necessary interruptions |  |
| Playing with mobile phone/taking incoming calls |  |
| Negative statements about one's own performance (e.g.: "I can't do this", "I'll never be able to do this", "I don't want to do this anymore") |  |
| Positive comments on the assessment (e.g. "I enjoyed it", "I find it interesting") |  |
| Inquiries about test performance |  |
| Patient had to be encouraged to continue |  |

|  | | **During the assessment** | | **Aplies fully** | | **Rather applies** | | **Barely apply** | **Does not apply at all** | | | **Cannot be assessed** | |
| --- | --- | --- | --- | --- | --- | --- | --- | --- | --- | --- | --- | --- | --- |
| 1. | | Patient seemed anxious about his/her performance. | | 1 | | 2 | | 3 | 4 | | | 0 | |
| 2. | | Patient seemed nervous (e.g. biting fingernails). | | 1 | | 2 | | 3 | 4 | | | 0 | |
| 3. | | Patient was afraid to make mistakes. | | 1 | | 2 | | 3 | 4 | | | 0 | |
| 4. | | Patient was partly worried about the tasks (if so, which procedures): _________________. | | 1 | | 2 | | 3 | 4 | | | 0 | |
| 5. | | Patient often sought reassurance regarding performance. | | 1 | | 2 | | 3 | 4 | | | 0 | |
|  | | | | **Aplies fully** | | **Rather applies** | | **Barely apply** | | | **Does not apply at all** | **Cannot be assessed** | |
| 6. | | Patient suffered from hallucinations during the tests. | | 1 | | 2 | | 3 | | | 4 | 0 | |
| 7. | | Patient displayed delusional thoughts during the tests (e.g. being observed/followed). | | 1 | | 2 | | 3 | | | 4 | 0 | |
| 8. | | Patient was extremely suspicious of the examiner/procedure during the assessment. | | 1 | | 2 | | 3 | | | 4 | 0 | |
| 9. | | Patient suffered from obsessive thoughts, brooding or depressive thoughts during the assessment. | | 1 | | 2 | | 3 | | | 4 | 0 | |
| 10. | | Patient performed compulsions during assessment. | | 1 | | 2 | | 3 | | | 4 | 0 | |
| 11. | | Patient spoke of cancelling the assessment. | | 1 | | 2 | | 3 | | | 4 | 0 | |
| 12. | | Patient was tired quickly, yawned a lot. | | 1 | | 2 | | 3 | | | 4 | 0 | |
| 13. | | Patient needed several breaks (number: ________________). | | 1 | | 2 | | 3 | | | 4 | 0 | |
| 14. | | Patient seemed bored. | | 1 | | 2 | | 3 | | | 4 | 0 | |
| 15. | | Patient often needed to be encouraged/motivated. | | 1 | | 2 | | 3 | | | 4 | 0 | |
| 16. | | Patient was unmotivated. | | 1 | | 2 | | 3 | | | 4 | 0 | |
| 17. | | Patient expressed having fun. | | 1 | | 2 | | 3 | | | 4 | 0 | |
| 18. | | Patient showed interest (e.g., inquiries about the background of the test). | | 1 | | 2 | | 3 | | | 4 | 0 | |
| 19. | | Patient was annoyed by his/her poor performance. | | 1 | | 2 | | 3 | | | 4 | 0 | |
| 20. | | Patient showed significant lapses in attention. | | 1 | | 2 | | 3 | | | 4 | 0 | |
| 21. | | Patient was very tense. | | 1 | | 2 | | 3 | | | 4 | 0 | |
| 22. | | Patient was quickly distracted/internally distracted by thoughts. | | 1 | | 2 | | 3 | | | 4 | 0 | |

|  | | Not applicable | applicable |
| --- | --- | --- | --- |
| 23. | Patient was on the phone during the assessment/wrote SMS. | 0 | 1 |
| 24. | Patient made remarks like "I don't like to be tested". | 0 | 1 |

Well-Being During Assessment = items 1, 2, 3, 4, 5, 9, 19, 21

Motivation = items 12, 14, 15, 16, 20, 22
